# Use of *Wisteria Floribunda* Agglutinin-Positive Human Mac-2 Binding Protein in Assessing Risk of Hepatocellular Carcinoma Due to Hepatitis B Virus

**DOI:** 10.1097/MD.0000000000003328

**Published:** 2016-04-08

**Authors:** Ja Yoon Heo, Seung Up Kim, Beom Kyung Kim, Jun Yong Park, Do Young Kim, Sang Hoon Ahn, Young Nyun Park, Sung Soo Ahn, Kwang-Hyub Han, Hyon-Suk Kim

**Affiliations:** From the Department of Internal Medicine (JYH, SUK, BKK, JYP, DYK, SHA, SSA, K-HH); Institute of Gastroenterology (SUK, BKK, JYP, DYK, SHA, K-HH); Department of Laboratory Medicine (H-SK); and Department of Pathology (YNP), Yonsei University College of Medicine, Seoul, South Korea.

## Abstract

*Wisteria floribunda* agglutinin-positive human Mac-2 binding protein (WFA^+^-M2BP) is a serologic marker corresponding with degree of hepatic fibrosis. We evaluated its accuracy in assessing hepatic fibrosis and in predicting the risk of developing hepatocellular carcinoma (HCC) in patients with chronic hepatitis B (CHB).

In a 5-year period (2009–2013), a total of 95 CHB patients with available serum WFA^+^-M2BP assay and transient elastography assessment [to assess liver stiffness (LS)] who had undergone liver biopsy were recruited for retrospective analysis.

Areas under the receiver operating characteristic curve for predicting fibrosis stages via serum WFA^+^-M2BP level were as follows: ≥F2, 0.688; ≥F3, 0.694; and F4, 0.704 (all *P* < 0.05). During the follow-up period (median, 45 months), HCC developed in 7 patients (7.4%). In patients with HCC, age, use of antiviral therapy, test parameters (HBV DNA, WFA^+^-M2BP, and LS determinations), and histologic stage of fibrosis were all significantly greater than in those free of HCC, whereas platelet count was significantly lower (all *P* < 0.05). On multivariate analysis, WFA^+^-M2BP was found independently predictive of emergent HCC [hazard ratio (HR) = 2.375; *P* = 0.036], although LS and histologic stage of fibrosis were not (*P* > 0.05). Risk of developing HCC was significantly greater in patients with high WFA^+^-M2BP levels (≥1.8) (adjusted HR = 11.5; *P* = 0.025). Cumulative incidence rates of HCC were also significantly higher in patients with high (vs. low) levels of WFA^+^-M2BP (log-rank test, *P* = 0.016).

WFA^+^-M2BP determination significantly reflected degree/extent of hepatic fibrosis and independently predicted the risk of developing HCC in patients with CHB.

## INTRODUCTION

Worldwide, it is estimated that 2 billion people been infected (past or present) with hepatitis B virus (HBV), and in 240 million, the infections are chronic.^1^ The clinical spectrum of HBV infection varies from subclinical inactive carrier state to progressive chronic hepatitis, culminating in cirrhosis, decompensation, and hepatocellular carcinoma (HCC).^2^ HBV infection constitutes the most frequent cause of mortality from cirrhosis and HCC, compared with any other viral and nonviral infection of liver, accounting for >780,000 deaths annually.^3^

During the past decade, new potent antiviral agents for chronic hepatitis B (CHB) viral infections have significantly prevented disease progression and reduced the risk of developing HCC.^4^ In addition, there is evidence that HBV-induced hepatic fibrosis can be reversed by prolonged antiviral therapy.^5–7^ Nevertheless, advanced fibrosis or cirrhosis still remains the single-most important marker of poor long-term prognosis, including emergence of HCC.^8^ Hence, gauging the degree of hepatic fibrosis is still an essential element in designing individualized surveillance strategies to detect HBV-related HCC in this era of antiviral treatment.^9^

To date, liver biopsy (LB) remains the gold standard for evaluating extent of hepatic fibrosis.^10^ However, LB is expensive, requires expertise, and is subject to procedural complications, including pain, bleeding, perforation, and even death. In addition, sampling error and interpretational variability are acknowledged as potential limitations.^11^ A number of noninvasive surrogate methods subsequently have been devised to surmount these shortcomings. Physical tests, such as transient elastography (TE) and acoustic radiation force impulse (ARFI), and serologic markers, including FibroTest (FT), enhanced liver fibrosis (ELF) panel, and aspartate aminotransferase-to-platelet ratio (APRI), are currently advocated as alternatives to LB.^12^

More recently, a glycan-based immunoassay has been introduced, targeting WFA^+^-M2BP as a biomarker for noninvasive gauging of hepatic fibrosis.^13^ Briefly, M2BP is secreted from many cell types, including hepatocytes, and its fluctuations during N-glycosylation mirror the progression of liver disease.^13^ Results of a recent study by Toshima et al^14^ indicate that WFA^+^-M2BP serum levels accurately reflect degrees of hepatic fibrosis, with an area under the receiver operating characteristic curve (AUC) of 0.812 in determining an advanced histologic stage (F ≥3). Another recent longitudinal follow-up study by Yamasaki et al^15^ disclosed a significant association between serum WFA^+^-M2BP level and the risk of emergent HCC in the context of chronic hepatitis C viral infection. In addition, WFA^+^-M2BP is reported to be a reliable marker of hepatic fibrosis and prognosis in patients with primary biliary cirrhosis and nonalcoholic fatty liver disease.^16,17^

Because serum WFA^+^-M2BP has performed reliably as a noninvasive biomarker in other chronic liver diseases, this single-center, retrospective cohort study was conducted to evaluate its accuracy in gauging hepatic fibrosis and in predicting the risk of developing HCC due to chronic HBV infection.

## METHODS

### Patients

In a 5-year period (2009–2013), 95 CHB patients with available stored serum sample at the time of LB and TE were eligible for this study. CHB was defined as persistence of serum HBV surface antigen for >6 months and HBV DNA-positivity by polymerase chain reaction assay. With written informed consent, serum samples taken at LB and TE were stored in the Yonsei Liver Blood Bank (YLBB) system (approval number, 4-2009-0725). The biopsies were done to assess severity of hepatic fibrosis and intensity of inflammation before starting antiviral therapy.

Exclusion criteria were as follows: LS measurement failure (valid shot = 0); invalid LS value; existing HCC at enrollment (or history thereof); HCC development within 6 months after enrollment; history of antiviral therapy, decompensated cirrhosis, or past/present cancers other than HCC; Child–Pugh class B or C at enrollment; LB unsuitable for proper interpretation; stored serum sample unsatisfactory for WFA^+^-M2BP assay; and coinfection with hepatitis C virus (HCV) or HIV.

This study was performed in accordance with ethical guidelines of the 1975 Declaration of Helsinki and approved by the Institutional Review Board of Severance Hospital. Given its retrospective nature, written informed consent was not required to access clinical data.

### Histologic Evaluation

LB specimens were fixed in formalin, processed routinely, and embedded in paraffin. Standard hematoxylin and eosin (H&E) and trichrome (Masson) stains were then performed, using 4-μm sections. All liver tissue samples were evaluated by an experienced pathologist blinded to patient clinical data, including TE results and serum WFA^+^-M2BP levels. Liver histology was scored semi-quantitatively, according to Batts and Ludwig criteria.^18^ Fibrosis was staged (0–4) as follows: F0, no fibrosis; F1, portal fibrosis without septa; F2, portal fibrosis and few septa; F3, numerous septa without cirrhosis; and F4, cirrhosis.

### Measurement of Serum WFA^+^-M2BP

Serum WFA^+^-M2BP was quantified by lectin-Ab sandwich immunoassay, using a fully automated immunoanalyzer (HISCL-2000i; Sysmex Co, Hyogo, Japan).^19^ Measured analyte levels, conjugated to *Wisteria floribunda* agglutinin, were indexed using the following equation: 
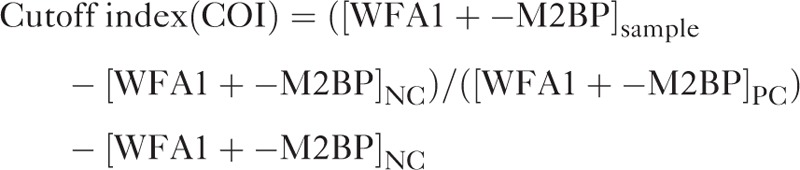


where [WFA1+-M2BP]_sample_ is WFA^+^-M2BP level in serum, PC is positive control, and NC is negative control. The positive control was supplied as a calibration solution preliminarily standardized to yield a cut-off index (COI) of 1.0.^19^

### LS Measurement by TE

Patients underwent TE at the time of enrollment by a well-trained technician. Both technique and examination procedure have been mentioned in previous literatures.^20–23^ Results were expressed as kilopascals (kPa). Interquartile range (IQR), as an index of intrinsic LS variability by definition, corresponded with the LS determinant interval containing 50% of valid procurements between 25th and 75th percentiles. Only LS values with a minimum 10 validated measurements and a 60% success rate were reliably included in this study. The median of successfully measured values was presumed representative of LS in a given patient only at IQR-to-median value ratios (IQR/M) of <0.3.

### Baseline Workup and Follow-Up

Enrollment baseline was the date of LB and TE procurement. At enrollment, all patients were evaluated to confirm the absence of HCC using ultrasonography and laboratory tests, including alpha-fetoprotein (AFP). If a given patient had HCC at enrollment, the patient was excluded for this study. After enrollment, all patients were followed up at every 3- to 6- month intervals for HCC surveillance and screening for liver-related complications. Diagnosis of HCC was based on guidelines proposed by the Korea Liver Cancer Study Group.^24^ Accordingly, a patient having 1 or more risk factors (hepatitis B or C viral infection, cirrhosis), with either serum AFP >400 ng/mL and positive findings on at least 1 of 3 customary imaging studies [dynamic computed tomography (CT), dynamic magnetic resonance imaging (MRI), or hepatic angiography] or serum AFP <400 ng/mL and positive findings on at least 2 of 3 imaging studies, was considered positive for HCC. On dynamic CT or MRI, increased arterial enhancement followed by diminished enhancement relative liver (washout) in portal or equilibrium phase was the diagnostic hallmark of HCC.

### Statistical Analysis

All data were expressed as median (IQR) or n (%), where indicated. Student *t* test (or Mann–Whitney test) and Chi-squared test (or Fisher's exact test) were utilized to examine the statistical significance of differences among continuous and categorical variables. The accuracy of determinants (WFA^+^-M2BP or LS) in depicting degree of liver fibrosis was indicated by the area under receiver operating characteristics curve (AUC), coupled with a 95% confidence interval (CI). Cutpoints were determined to maximize the sum of sensitivity and specificity from receiver operating characteristic curve analyses and corresponding diagnostic indices were calculated. The DeLong method was used to compare AUC values of WFA^+^-M2BP and LS. For longitudinal analysis, patients were censored at time of HCC detection or at last follow-up. Cumulative HCC incidence rates were estimated via Kaplan–Meier method and compared using log-rank test. Independent risk factors for HCC development were identified by univariate and subsequent multivariate Cox regression analyses. Hazard ratios (HRs) and corresponding 95% CIs were calculated. All statistical analyses relied on standard software (SPSS v18.0; SPSS Inc, Chicago, IL), with *P* value <0.05 considered statistically significant.

## RESULTS

### Patient Characteristics

On the basis of our exclusion criteria, 95 patients with CHB were recruited for study, all previously undergoing LB, TE, and serum WFA^+^-M2BP assay. Baseline characteristics of the patient population are summarized in Table 1. Median age was 51 (IQR, 44–60) years; 69 patients (72.6%) were men; and 78 patients (82.1%) received antiviral therapy at enrollment or during follow-up. Median values of alanine aminotransferase (ALT), serum albumin, total bilirubin, platelet count were 41 (IQR, 28–66) IU/L, 4.2 (IQR, 3.9–4.4) g/dL, 0.7 (IQR, 0.6–0.9) mg/dL, and 171 (IQR, 135–220) 10^9^/L, respectively. Fifty-one patients (53.7%) tested positive for HBV e antigen (HBeAg), and in 63 patients (66.3%), HBV DNA levels were >10^5^copies/mL.

**TABLE 1:**
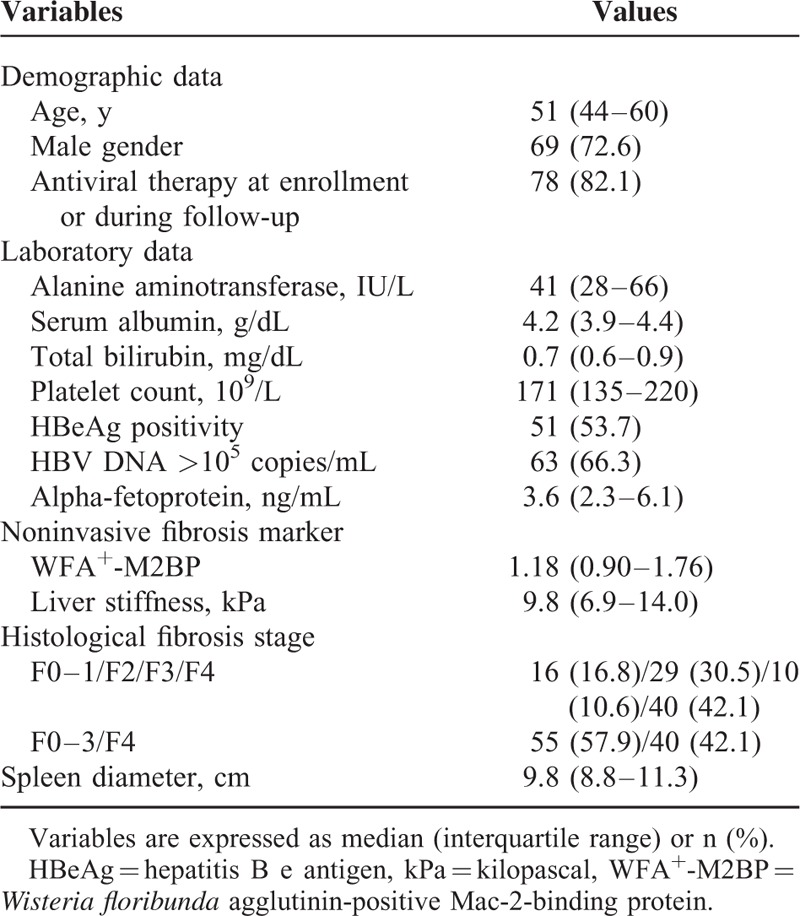
Baseline Characteristics (n = 95)

Median WFA^+^-M2BP and LS values were 1.18 (IQR, 0.9–1.76) and 9.8 (IQR, 6.9–14.0) kPa, respectively. In study participants, histological fibrosis staging was as follows: F1, 16 (16.8%); F2, 29 (30.5%); F3, 10 (10.6%), and F4, 40 (42.1%).

### Diagnostic Test Performance (WFA^+^-M2BP and LS)

Performances of WFA^+^-M2BP, LS, and their combinations are summarized in Table 2. The AUC values for WFA^+^-M2BP in predicting fibrosis stage ≥F2, ≥F3, and F4 were 0.688 (95% CI, 0.539–0.836), 0.694 (95% CI, 0.589–0.798), and 0.704 (95% CI, 0.599–0.810), respectively (all *P* < 0.05). Optimal cutpoints were 0.8 for ≥F2 [sensitivity, 87.3%; specificity, 43.8%; positive predictive value (PPV), 80.0%; negative predictive value (NPV), 41.2%], 1.6 for ≥F3 (sensitivity, 36.0%; specificity, 88.9%; PPV, 78.3%; NPV, 55.6%), and 2.0 for F4 (sensitivity, 35.0%; specificity, 92.7%; PPV, 77.8%; NPV, 66.2%).

**TABLE 2:**
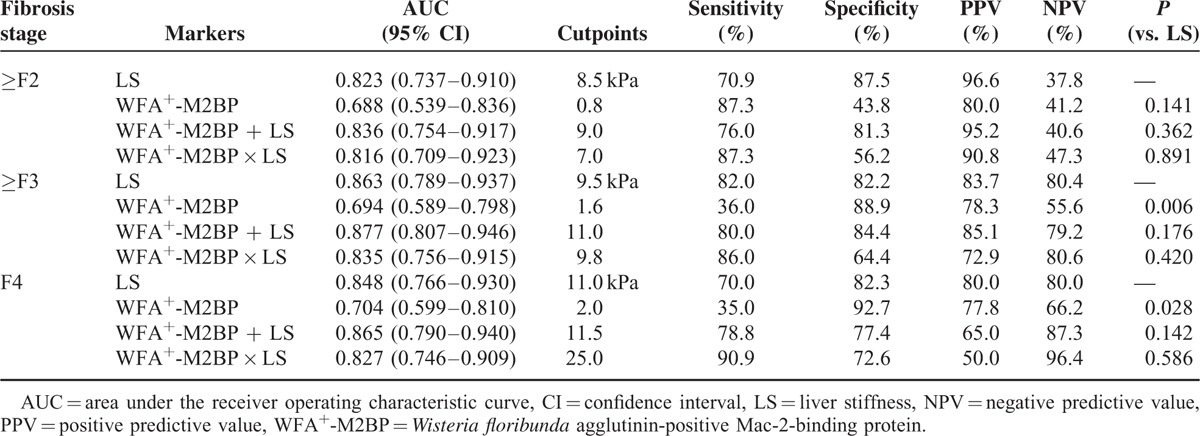
Diagnostic Performance of WFA^+^-M2BP and LS and Their Combinations

With respect to LS, AUC values were 0.823 (95% CI, 0.737–0.910), 0.863 (95% CI, 0.789–0.937), and 0.848 (95% CI, 0.766–0.930), respectively. Optimal cutpoints were 8.5 kPa for ≥F2 (sensitivity, 70.9%; specificity, 87.5%; PPV, 96.6%; NPV, 37.8%), 9.5 kPa for ≥F3 (sensitivity, 82.0%; specificity, 82.2%; PPV, 83.7%; NPV, 80.4%), and 11.0 kPa for F4 (sensitivity, 70.0%; specificity, 82.3%; PPV, 80.0%; NPV, 80.0%).

In terms of accuracy, LS was significantly superior to WFA^+^-M2BP in predicting stages ≥F3 and F4 stage (both *P* < 0.05), although stages ≥F2 were similarly predicted (*P* = 0.141). The accuracy of combining WFA^+^-M2BP and LS was similar to that of LS in predicting all fibrosis stages (all *P *> 0.05) (Table 2).

### Comparison of Baseline Characteristics in Patients With and Without HCC

Baseline characteristics of patients with and without HCC are summarized in Table 3. Age (median, 62 vs. 51 years), proportionate use of antiviral therapy (100 vs. 74.7%), HBV DNA level >10^5^ copies/mL (100.0 vs. 58.9%), serum WFA^+^-M2BP level (median, 2.2 vs. 1.1), LS value (median, 16.8 vs. 8.9 kPa), and proportion with F4 stage fibrosis (85.7 vs. 35.8%) were significantly greater in patients who developed HCC, than those who did not (all *P* < 0.05), whereas platelet count (median 133 vs. 177 10^9^/L) was significantly lower by comparison (*P* < 0.05).

**TABLE 3:**
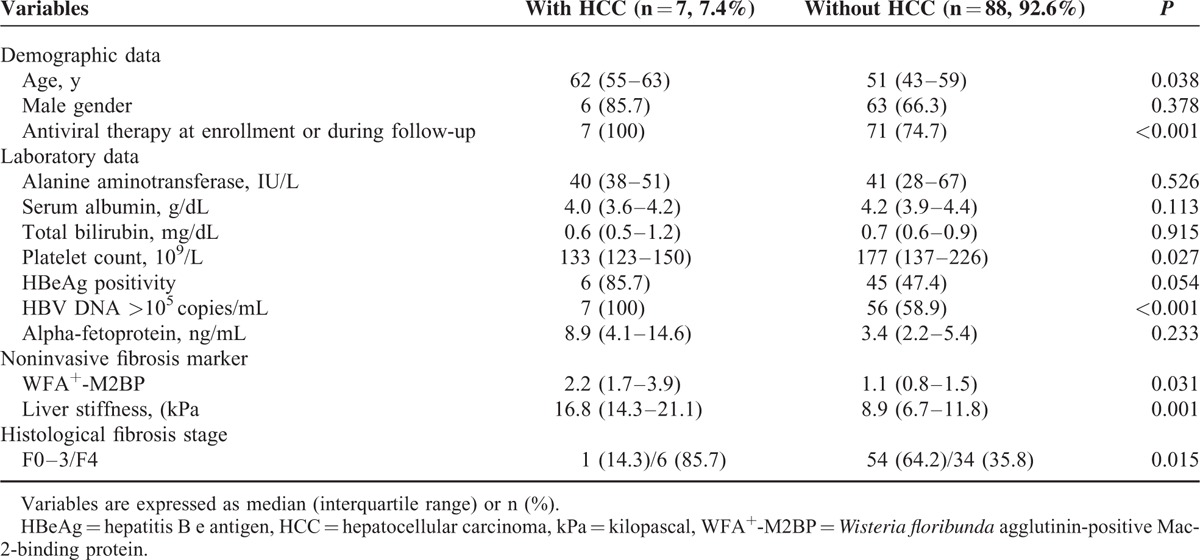
Comparison Between Patients With and Without HCC Development

### Factors Associated With HCC Development

To prevent statistical colinearity, WFA^+^-M2BP, LS, and histologic stage of fibrosis were incorporated into respective multivariate analyses (Table 4). Platelet count, WFA^+^-M2BP, LS, and fibrosis stage displayed significance in univariate analysis (all *P* < 0.05). However, WFA^+^-M2BP (after adjustment) emerged as the sole independent predictor of developing HCC (HR = 2.375, 95% CI 1.056–5.340; *P* = 0.036), with platelet count showing borderline statistical significance (*P* = 0.059). With adjustment of platelet count and LS, platelet count did show statistical significance (HR = 0.972, 95% CI 0.947–0.997; *P* = 0.031), whereas LS was borderline (*P* = 0.065). Only platelet count proved significant (HR = 0.971, 95% CI 0.946–0.997; *P* = 0.027) with adjustment of fibrosis stage.

**TABLE 4:**
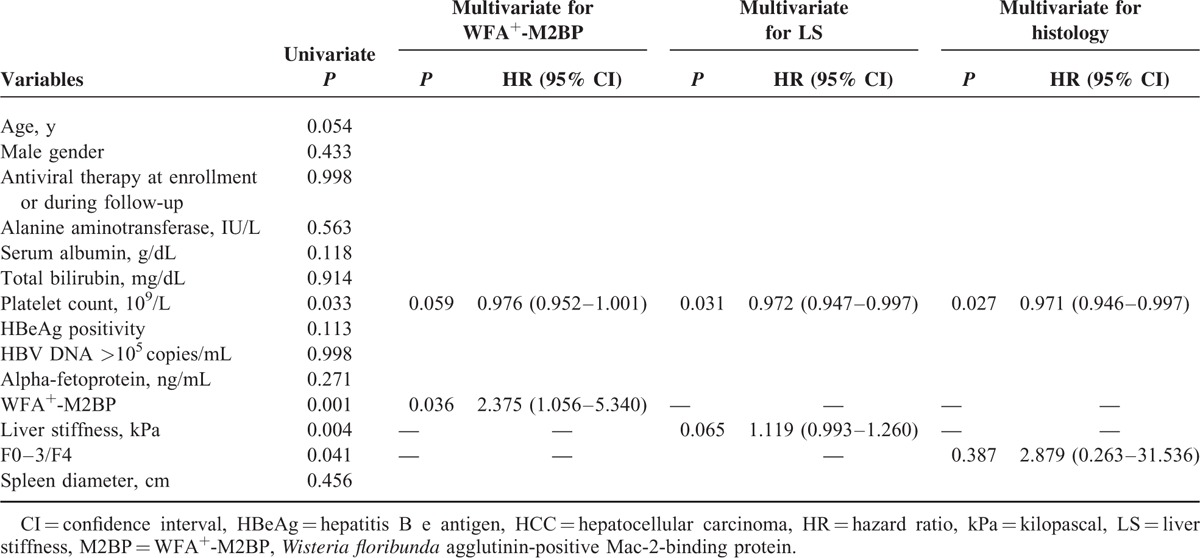
Independent factors associated with HCC development

### Relative Risk of Developing HCC With Binary Stratification

**FIGURE 1:**
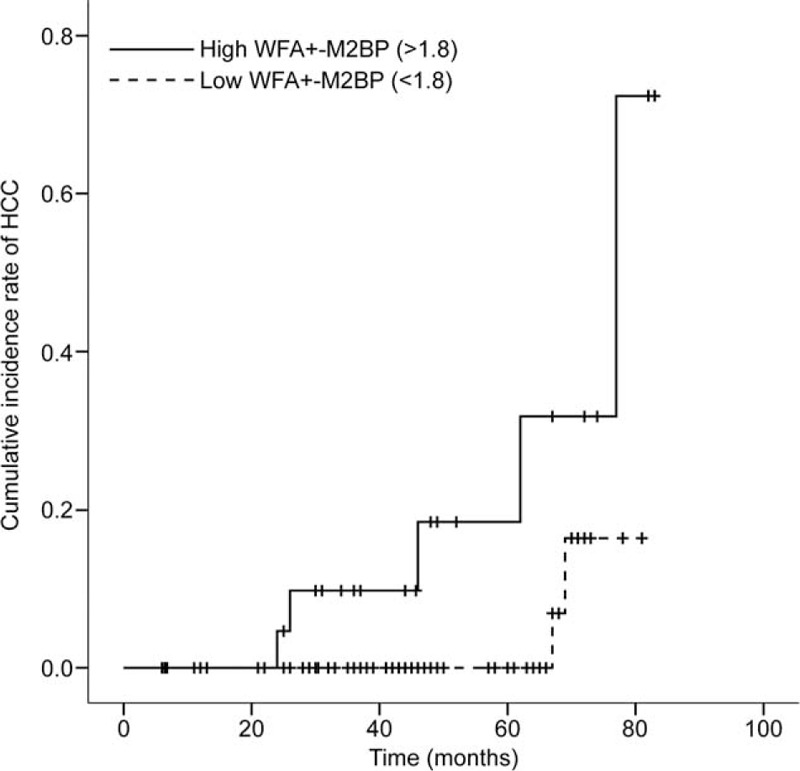
Cumulative rates of HCC in 2-tier stratification of serum WFA^+^-M2BP levels (Kaplan–Meier plot). Risk increased significantly with corresponding increase in WFA^+^-M2BP levels between groups (*P* = 0.016, log-rank test).

Table 5 summarizes the relative risk of developing HCC in binary stratified subsets. Due to small sample size and no instances of HCC among patients with serum levels of WFA^+^-M2BP less than median value (1.18), the third quartile value of 1.80 served as cutpoint of WFA^+^-M2BP levels in binary stratification, thus calculating the relative risk of emergent HCC in patients with high (vs. low) WFA^+^-M2BP levels. Of the 2 groups, assigned by serum WFA^+^-M2BP levels (<1.80 vs. ≥1.80), the subset with high levels (≥1.80) showed significantly greater risk of developing HCC by comparison (HR = 12.8, 95% CI 1.5–107.0; *P* = 0.019). Cumulative incidence rates of HCC were also significantly higher in patients with high (vs. low) levels of WFA^+^-M2BP (*P* = 0.016, log-rank test) (Figure 1). With adjustment of platelet count, the higher relative risk of patients with high levels of WFA^+^-M2BP was maintained (HR = 1.5, 95% CI 1.4–97.2; *P* = 0.025). In comparing risk at F4 stage fibrosis with risk at lesser stages (F0-F3), statistical significance was not reached (all *P* > 0.05).

**TABLE 5:**
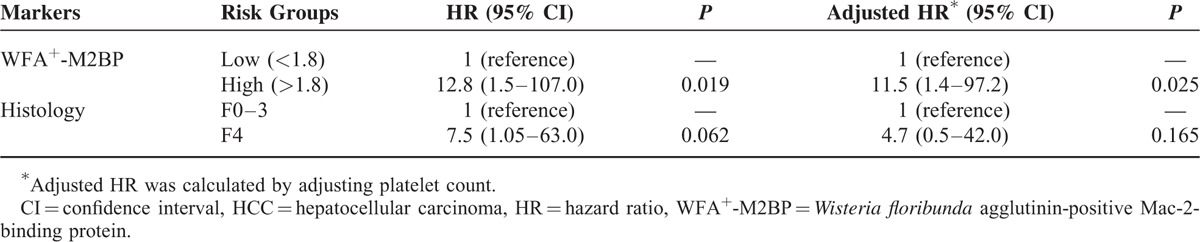
Relative Risk of HCC Development According to Binary Stratification

## DISCUSSION

In this era of potent HBV antiviral therapy, the burden of fibrosis is considered the single-most important risk factor for developing HCC due to HBV infection.^8^ Because tissue samplings for evaluating fibrosis are not always feasible, various noninvasive physical and biochemical tests (i.e., TE, ARFI, FT, ELF, and APRI) have been developed. Recently, M2BP, a protein involved in cell adhesion, was found to vary in quality and quantity during progressive fibrosis.^25,26^ Activation of hepatic stellate cells and reversal of such activation may coincide with shifts in WFA^+^-M2BP levels.^27^ However, it is apparent that WFA^+^-M2BP more specifically reflects the progression of fibrosis and thus is a suitable novel surrogate for LB in chronic liver diseases.^19,28^

In this study, the diagnostic accuracy of serum WFA^+^-M2BP assay at specified stages of fibrosis seemed unsatisfactory (AUC ∼0.7: ≥F2, 0.688, ≥F3, 0.694; F4, 0.704); and compared with LS determination, the diagnostic accuracy of serum WFA^+^-M2BP level in predicting stages ≥F3 or F4 stage was significantly inferior (both *P* < 0.05), despite similarity in predicting stages ≥F2 (*P* = 0.141). However, as the extent of fibrosis increased, specificity rose from 43.8 to 92.7%, implying that WFA^+^-M2BP may be useful in detecting advanced fibrosis or cirrhosis, regardless of the inferiority displayed overall. From a longitudinal perspective, serum WFA^+^-M2BP levels (similar to LS value) were significantly higher in patients prone to development of HCC, as opposed to those who were not (median, 2.2 vs. 1.1; *P* = 0.031). Furthermore, measured serum WFA^+^-M2BP proved independently predictive of HCC development (HR = 2.375), whereas histologic staging of fibrosis did not (*P* > 0.05). Finally, by stratifying the study population into 2 groups, based on a cutpoint of 1.8 for the WFA^+^-M2BP marker, a significantly greater risk (adjusted HR = 11.5) of developing HCC at high (vs. low) levels of WFA^+^-M2BP was evident. For these reasons, serum WFA^+^-M2BP level may reasonably be used to assess risk of developing HCC in patients with CHB.

Our study has several strong points. Although past efforts have documented the accuracy of WFA^+^-M2BP assay in patients with chronic liver diseases of other etiologies, none has yet addressed HBV. In HCV infection, for instance, AUC values reported by stage of fibrosis were 0.83 (≥F2) and 0.74 (≥F3),^29^ and in primary biliary cirrhosis, AUC values cited were 0.979 (≥F2), 0.933 (≥F3), and 0.965 (F4).^17^ A significant association between WFA^+^-M2BP level and fibrotic burden was also established in patients with nonalcoholic fatty liver disease (AUC: 0.876, ≥F2; 0.879, ≥F3).^16^ Moreover, in autoimmune hepatitis, the AUC of WFA^+^-M2BP in predicting stage F4 fibrosis was 0.853, exceeding that of other serologic fibrosis markers, including FIB-4 index and hyaluronic acid.^30^ Thus, our findings offer a basis for future large-scale validation studies directed at HBV.

Through TE examination, we also pursued LS in the context of HBV as a marker of hepatic fibrosis, demonstrating its greater accuracy relative to serum WFA^+^-M2BP.^20,22,23,31–34^ In our cross-sectional analysis, LS value showed accuracy similar to that encountered in prior studies (AUC: 0.823–0.848),^23,35^ suggesting that even with the limited size of this cohort, patient characteristics were appropriate for evaluating WFA^+^-M2BP as well. However, the diagnostic accuracy of WFA^+^-M2BP (AUC ∼0.7) proved unsatisfactory, compared with other studies (AUC >0.8). Reasons for this are unclear but may in part be explained by inherent properties of HBV, in that background fibrosis often fluctuates during the course of disease.^36^ On the contrary, HCV, primary biliary cirrhosis, and autoimmune hepatitis are more insidious in nature, resulting in slowly progressive fibrosis.^37,38^ Probably due to this unsatisfactory accuracy of M2BP, the combination of WFA^+^-M2BP and LS could not enhance the overall accuracy in predicting all fibrosis stages in comparison to LS (all *P* > 0.05). Apart from cross-sectional analysis, which appeared unsatisfactory, we similarly explored the prognostic value of WFA^+^-M2BP from a longitudinal aspect. Serum WFA^+^-M2BP levels proved significantly predictive in terms of risk involved in developing HCC. Previous studies likewise have concluded that serum WFA^+^-M2BP levels may reflect pre-cancer status or hepatocellular carcinogenesis, rather than a product of existing HCC,^39^ and are predictive of HCC arising in patients chronically infected with HCV.^15,39,40^ Consequently, a prospective large-scale study should be conducted to validate our results, however questionable the cross-sectional analysis.

We are also aware of several unresolved issues in our study. First, our sample size was small, and instances of HCC were few. Furthermore, longitudinal analysis of LS indicated only borderline statistical significance in assessing the risk of developing HCC (*P* = 0.065), despite notable similarity to previous TE-based investigations (AUC >0.8).^23,35^ Given that LS value is a known factor in predicting the risk of HCC,^21,31,32^ this seemingly inconsistent outcome may be related to the limited sampling. Sample size was also inadequate to systematically control relevant variables, such as antiviral agent use, existing cirrhosis, and necroinflammatory activity, for subgroup analyses, even though the appropriate adjustments were made in multivariate analyses. In addition, only binary stratification of the study population was feasible, rather than a detailed step-wise delineation according to WFA^+^-M2BP level, thus determining relative risks among subgroups. Larger populations are needed to substantiate present findings. Another issue is that our serologic cutpoints for WFA^+^-M2BP differed from those of prior studies [0.8 (≥F2) vs. 0.90–1.86; 1.6 (≥F3) vs. 0.94–2.21; 2.0 (F4) vs. 1.46–2.64]. However, this was not the case with cutpoints determined for LS values (confirmed by other publications),^14,16,17,29^ so it is possible that disease etiology, and not sample size, is pivotal in arriving at thresholds of WFA^+^-M2BP. Still, an optimal cutpoint for serum WFA^+^-M2BP awaits investigation in terms of HBV. Finally, we found it difficult to investigate whether WFA^+^-M2BP is useful for dynamic monitoring of HCC risk, detecting changes in fibrotic burden during prolonged antiviral treatment. Only baseline serum WFA^+^-M2BP levels were available. Because it is virtually impossible to serially sample the liver in routine practice, serial WFA^+^-M2BP assays may affirm the prognostic utility of this approach, a matter for future studies.

In conclusion, our study disclosed an independent longitudinal association between serum WFA^+^-M2BP level and the risk of emergent HCC in patients with CHB, despite its unsatisfactory performance in gauging fibrotic burden on cross-sectional analysis. Further large-scale study is warranted to validate the prognostic role of WFA^+^-M2BP during clinical surveillance of such patients.
